# Poster Session I - A135 EFFECT OF MUSIC THERAPY ON PATIENT EXPERIENCE IN GASTROINTESTINAL ENDOSCOPY: A SCOPING REVIEW

**DOI:** 10.1093/jcag/gwaf042.135

**Published:** 2026-02-13

**Authors:** J Hearn, S Carpentier

**Affiliations:** Gastroenterology, Queen’s University, Kingston, ON, Canada; Dalhousie University, Saint John, NB, Canada

## Abstract

**Background:**

Patient-reported outcome measures (PROMs) and patient-reported experience measures (PREMs) are simple and effective means of quantifying the patient experience and identifying ways in which we can provide more patient-centred care. Music therapy is a low-cost and low-risk intervention that has been shown to improve PROMs and PREMs in various areas of medicine. Gastrointestinal (GI) endoscopy presents an area in which music therapy could make a significant impact, particularly given the large number of these procedures performed each year and the pre-procedural anxiety often faced by patients.

**Aims:**

A scoping review was performed to answer the following research question: *What is known from the existing literature about the effect of music therapy used in adult GI endoscopy on PROMs (e.g. pain, anxiety) and PREMs (e.g. satisfaction, willingness to repeat the procedure)?*

**Methods:**

Guided by the methodological framework proposed by Arksey and O’Malley, three medical databases were queried for articles pertinent to the research question and published between January 2005 and December 2024. The implemented search strategy comprised three major themes: *music therapy, GI endoscopy* and *patient-reported measures.*

**Results:**

A total of 30 original research articles were selected for inclusion. Studies assessed the effect of music therapy in both lower endoscopy (N = 24) and upper endoscopy (N = 9). The most common patient-reported measures were pain (N = 21), anxiety (N = 21), and satisfaction (N = 14). Significant improvements following music therapy were described most commonly for anxiety (N = 15, 71% of 21) and satisfaction (N = 10, 71% of 14), and less commonly for pain (N = 11, 52% of 21) (**Figure 1**). Reductions in pain and anxiety were more consistent for music interventions implemented in the pre-endoscopy period.

**Conclusions:**

Music therapy is a simple, low-cost and low-risk intervention with the potential to improve the experience of patients undergoing GI endoscopy. The existing literature suggests a general effectiveness of music therapy in improving patient anxiety and satisfaction. A reduction in pain has also been reported in several studies; however, this effect appears to be less reliable. Given the potential benefits and minimal associated risks, endoscopists should consider music therapy as a non-pharmacologic adjunct to improve the patient experience in GI endoscopy.

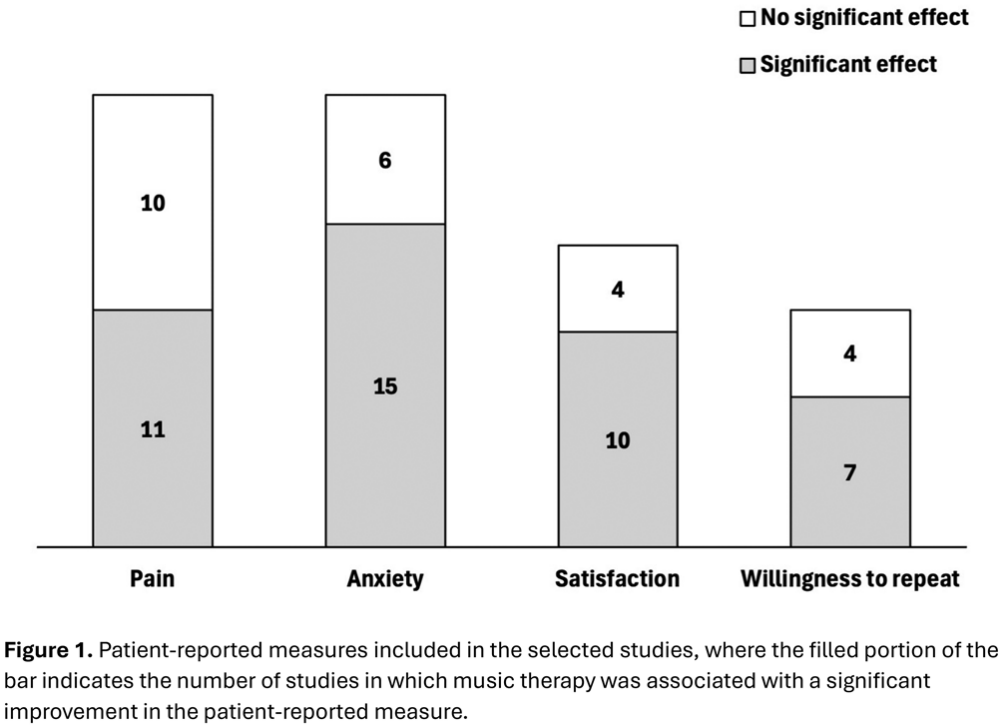

**Funding Agencies:**

None

